# Draft Whole-Genome Sequence of the Marine Bacterium *Idiomarina zobellii* KMM 231^T^

**DOI:** 10.1128/genomeA.01257-15

**Published:** 2015-10-29

**Authors:** Scott Mithoefer, Bruce A. Rheaume, Kyle S. MacLea

**Affiliations:** a Biology Program, University of New Hampshire, Manchester, New Hampshire, USA; b Biotechnology Program, University of New Hampshire, Manchester, New Hampshire, USA

## Abstract

*Idiomarina zobellii* was isolated from the northwest Pacific Ocean at a depth of 4,000 to 5,000 m in 1985. The draft whole-genome shotgun sequence of *I. zobellii* KMM 231^T^ described in this paper has a predicted length of 2,602,160 bp, containing 2,570 total genes, 52 tRNAs, and a G+C content of 47.10%.

## GENOME ANNOUNCEMENT

The bacterium *Idiomarina zobellii* strain KMM 231^T^ was originally collected in 1985 from the northwest Pacific Ocean at a depth of 4,000 to 5,000 m by Ivanova et al. (1). *I. zobellii* is a Gram-negative gammaproteobacterium bacillus, with fimbriae and a single long flagellum. It is heterotrophic and grows under strict aerobic conditions in extreme oceanic environments of low temperature, high salinity, and pressures between 397 and 497 atm (1).

*Idiomarina zobellii* strain KMM 231^T^ was obtained from ATCC (BAA-313) as a purified, freeze-dried sample and cultured in marine broth at a temperature of 30°C for 65 h at 1 atm. Genomic DNA extraction and isolation was performed using the Qiagen Genomic-tip 500/g kit (Valencia, CA, USA). The purified genomic DNA was fragmented and marked with adapters with the Nextera DNA Library prep kit (Illumina, San Diego, CA, USA), and the sequence library of 250-bp paired-end reads was generated on a HiSeq 2500 sequencer (Illumina) at the Hubbard Center for Genome Studies at the University of New Hampshire; 44,672,634 reads were produced and trimmed using Trimmomatic (2), and sequence assembly was achieved using the SPAdes genome assembler version 3.5.0 (3), which represented a 1,212.74-fold genome coverage when the contigs were analyzed with QUAST version 2.3 (4). Analysis of the genome assembly showed a length of 2,602,160 bp in 86 contiguous segments with a G+C content of 47.10% (4). Additionally, the *N*_50_ for the sequence was 248,687 bp, with the largest contiguous segment being 425,886 bp (4). Annotation of the sequence was accomplished through the NCBI Prokaryotic Genome Annotation Pipeline (5), which found 2,570 total genes of which 2,440 were coding sequences, 52 were tRNAs, and the balance were pseudogenes, rRNAs, and ncRNAs. A partial gene sequence for the 16S rRNA was noted, and comparison to the NCBI database through BLAST (6) revealed 100% similarity to previously deposited 16S rRNA sequences (e.g., DDBJ/ENA/GenBank accession number NR_024892) from *I. zobellii* strains, as well as close matches to other *Idiomarina* spp.

### Nucleotide sequence accession numbers. 

This whole-genome shotgun project has been deposited in DDBJ/ENA/GenBank under the accession number LHSG00000000. The version described in this paper is the first version, LHSG01000000.
